# Trends and hotspots in cupping therapy research for pain: a bibliometric study

**DOI:** 10.3389/fmed.2025.1559099

**Published:** 2025-07-09

**Authors:** Renjie Xu, Shi-Lu Liu, Guangxu Xu, Chengjie Yan, Qi Cui, Shan Liu, Mingliang Sun

**Affiliations:** ^1^Kunshan Rehabilitation Hospital, Suzhou, Jiangsu, China; ^2^School of Rehabilitation Medicine, Nanjing Medical University, Nanjing, Jiangsu, China; ^3^College School of Acupuncture-Moxibustion and Tuina, School of Health Preservation and Rehabilitation, Nanjing University of Chinese Medicine, Nanjing, China; ^4^Department of Rehabilitation Medicine, The Affiliated Suzhou Hospital of Nanjing Medical University, Suzhou, China; ^5^Department of Rehabilitation Medicine, The First Affiliated Hospital of Nanjing Medical University, Nanjing, Jiangsu, China; ^6^Shuyang Hospital of Traditional Chinese Medicine, Suqian, Jiangu, China

**Keywords:** cupping therapy, pain, CiteSpace, VOSviewer, visualization analysis, bibliometric

## Abstract

**Background:**

Cupping therapy (CT), a traditional form of alternative medicine, has gained attention as a non-pharmacological intervention for pain. Its applications span various pain-related conditions such as musculoskeletal disorders, migraines. Despite its growing popularity, comprehensive analyses of research trends, collaboration networks, and emerging hotspots in CT for pain remain limited.

**Methods:**

This bibliometric study analyzed 234 publications on CT for pain sourced from the Web of Science Core Collection. CiteSpace and VOSviewer software, and Bibliometric analysis website were employed to analyze trends, identify key contributors, and map global collaboration networks. Co-cited references, keyword clustering, and burst detection analyses were performed to uncover research hotspots and trends.

**Results:**

A total of 234 publications from 31 countries and 437 institutions were included. China led in publication volume, while the United States had the highest total citations. The Korea Institute of Oriental Medicine and the University of Duisburg-Essen were identified as central hubs for institutional collaboration. High-frequency keywords such as “pain,” “cupping therapy,” “acupuncture,” and “negative pressure” highlighted a focus on CT’s clinical applications and mechanisms. Emerging trends included the integration of CT with modalities like acupuncture and physical therapy. However, methodological limitations, such as inconsistent protocols and insufficient mechanistic studies, were identified as key challenges.

**Conclusion:**

This study offers an overview of the research landscape for CT in pain management, its potential as a safe and effective therapy. To strengthen its role in evidence-based medicine, future research should focus on standardizing treatment protocols, conducting high-quality clinical trials, and exploring its underlying mechanisms.

## 1 Introduction

Cupping therapy (CT), a traditional form of alternative medicine has gained increasing attention in recent decades as a potential treatment for pain management. CT utilizes the negative pressure of a cupping device to create suction on the skin to enhance blood flow, reduce inflammation and promote healing (1, 2). Clinically, two primary forms are distinguished. Dry cupping (also called retained or static cupping) employs negative pressure alone and leaves the skin intact (3), whereas wet cupping combines shallow scarification or puncture with suction to draw a small amount of blood, a practice often termed hijama in Middle Eastern medicine (4). While initially rooted in traditional medicine, CT has transitioned into modern clinical practice as part of integrative healthcare systems, particularly in the context of chronic pain conditions. The increasing global burden of pain-related disorders has driven researchers to explore non-pharmacological interventions like CT as complementary approaches to conventional pain management strategies (5). The increasing interest of researchers in CT is evidenced by its application in the treatment of a range of pain conditions, including musculoskeletal pain, migraines, and neuropathic disorders. Several studies (6–8) have indicated that this approach has the potential to alleviate pain, improve functional outcomes, and enhance the quality of life for patients suffering from chronic pain. CT is hypothesized to work through neurovascular and immune responses, as well as by reducing oxidative stress and stimulating endorphin release (9–12). The precise therapeutic mechanism of this treatment remains a topic of ongoing investigation, underscoring the necessity for rigorous evidence-based studies to substantiate its efficacy and safety. Bibliometric analysis provides a valuable method for comprehensive examination of existing research, identifying key trends, and discovering hotspots in specific fields (13). A recently published bibliometric overview of CT interventions for general health outcomes (14) provides a broad landscape but does not concentrate on pain-related evidence, and a survey of acupuncture for microcirculation and hemorheology included CT only as an ancillary technique without analyzing it independently (15). Consequently, the literature still lacks a dedicated synthesis of CT for pain. This study aims to address this gap by conducting a comprehensive bibliometric analysis of global research on CT for pain. Using data from the Web of Science Core Collection database (WoSCC), we seek to map research trends, identify high-impact publications, and explore key hotspots and collaboration networks. To provide a clearer understanding of the use of CT in pain management and the trajectory of CT in the broader context of complementary medicine. The findings of this study will not only serve as a reference for future research but also support healthcare practitioners in making decisions about integrating CT into clinical practice.

## 2 Methods

### 2.1 Ethic statement

This study is based entirely on the analysis of secondary literature, ethical approval was not required.

### 2.2 Data acquisition and retrieval strategy

**FIGURE 1:**
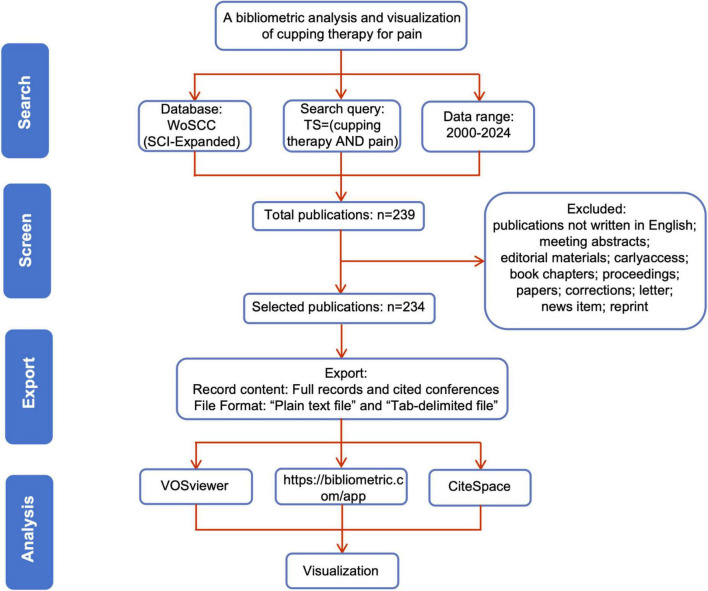
Flowchart of literature screening.

The data for this bibliometric analysis were extracted from the Science Citation Index Expanded (SCI-Expanded) database within WoSCC. The detailed steps for the literature search and screening are illustrated in Figure 1. The search was performed using the query: “TS = (cupping therapy) AND TS = (pain).” Only articles published in English were included in the final dataset. To avoid potential biases caused by regular database updates, the search was conducted on a predetermined date. A total of 234 publications, including research articles and review articles, were retrieved. The retrieved data were saved as complete records with cited references and exported in “Plain text file” and “Tab-delimited file” formats for subsequent analysis.

### 2.3 Data analysis

The eligible publications were exported as a plain text file titled “download_xxx.txt,” which included complete records and cited references. This file was imported into VOS viewer 1.6.19 and CiteSpace 6.2.R2 software to create knowledge maps and perform statistical analyses. Concurrently, the tab-delimited files were uploaded to an online platform for bibliometric visualization, aimed at constructing collaborative network maps for countries and regions. The VOS viewer software was configured with the following settings: association strength was used as the normalization method, and the minimum publication thresholds for country/region, institution, and author were set at 1, 2, and 3, respectively. Keywords were analyzed based on occurrence frequency, with a threshold set to 3.

The CiteSpace software parameters were set as follows: the analysis time frame spanned from January 2000 to December 2024, with the data divided into 1 year time slices. The analysis included nodes representing keywords and references, with the g-index value set at *k* = 25 for each time slice. Visualization was optimized by applying pruning techniques, including pathfinder, sliced network pruning, and merged network pruning. All other parameters were left at default settings.

This study evaluates various aspects of CT research for pain, including the volume of publications, contributions from countries, institutions, and authors, and journal and reference co-citation analyses. Furthermore, keyword co-occurrence and clustering analyses were employed to identify research hotspots. Finally, burst keyword analysis was used to pinpoint emerging research trends in this field.

## 3 Results

### 3.1 Annual publications and citations

**FIGURE 2:**
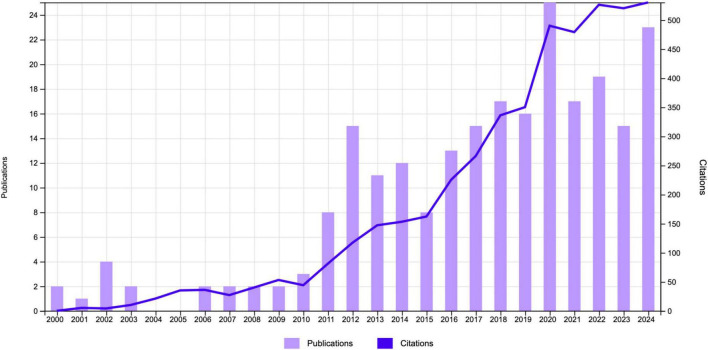
Annual publications and citations trend chart.

The study encompassed related publications relevant publications including 234 original research articles (77%) and 205 review articles (23%). The total number of citations (TC) was 3,296. The average number of citations per publication (ACPP) was 19.93 with 37 *h*-index. The annual trends in publications and citations related to CT for pain are illustrated in Figure 2. From 2000 to 2024, the field experienced a gradual yet significant growth in academic interest and scholarly contributions. During the early years (20,002,010), the number of publications remained relatively low, with less than five publications annually. This indicates that research on this topic was in its nascent stages. From 2011 onward, the number of annual publications began to rise steadily, reflecting a growing recognition of CT as a potential treatment for pain. The period between 2014 and 2020 marked a significant phase of growth, as annual publications consistently exceeded 15, with a peak in 2020. This surge may be attributed to the increasing integration of complementary and alternative therapies into pain management. Similarly, the number of citations exhibited an upward trajectory. While TC remained relatively modest prior to 2010, a gradual increase was observed following 2011. By 2023, the annual TC had surpassed 500, underscoring the broader impact and relevance of research in this field. The concurrent rise in publication volume and citation frequency during this period suggests that the field has transitioned into a mature phase of scientific inquiry, characterized by more robust research outputs. The combined trend of increasing publications and citations underscores the expanding academic focus on CT for pain management.

### 3.2 Distribution of countries/regions and institutions

**FIGURE 3:**
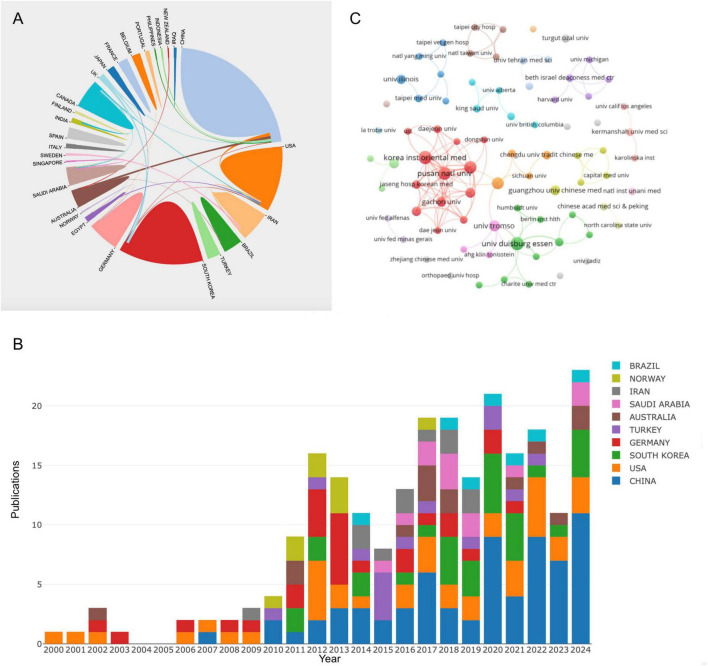
**(A)** National/regional collaborative network knowledge map. **(B)** Stack bar plot of top 10 countries/regions in publication. **(C)** Collaborative network knowledge map of institution.

This study analyzed 234 publications produced by 437 institutions across 31 countries. As shown in Table 1, Top 10 countries/regions ranked by number of publications were identified as significant contributors to the field of CT research for pain. China leads with 57 publications, 997 TC, and 16 total link strength (TLS) followed by the United States with 39 publications, 1,256 TC, and 15 TLS. South Korea ranks third with 30 publications, 270 TC, and 4 TLS. while Germany, with 27 publications, achieved a high ACPP of 37.04, reflecting high-impact research. Other contributors include Australia, Turkey, Saudi Arabia, and Iran, with Norway standing out for its high ACPP of 34.33 from 9 publications. Figure 3A illustrates the global collaboration networks among countries and regions, with colored blocks representing individual contributors. The size of each block corresponds to the number of publications, while the connecting lines indicate the strength of collaborative efforts, with thicker lines representing stronger relationships. China, the United States, South Korea, and Germany have actively collaborated with other countries, forming the core of the global research network. Figure 3B shows early contributions were dominated by a few countries, but China has led since 2012, reflecting its integration of traditional medicine with modern science. The United States and South Korea have consistently contributed, emphasizing evidence-based research and the integration of traditional medicine into healthcare systems.

**TABLE 1:** Top 10 countries/regions ranked by number of publications.

Rank	Countries	Publications	TC	ACPP	TLS	Centrality
1	China	57	997	17.49	16	0.17
2	The United States	39	1,256	32.21	15	0.37
3	South Korea	30	270	9	4	0
4	Germany	27	1,000	37.04	11	0.09
5	Australia	14	420	30	11	0.17
6	Turkey	14	124	8.86	0	0
7	Saudi Arabia	12	290	24.17	8	0.11
8	Iran	11	195	17.73	4	0
9	Taiwan	11	133	12.09	4	0.17
10	Norway	9	309	34.33	11	0.34

TC, total citations; ACPP, average number of citations per publication; TLS, total link strength.

Figure 3C presents the institutional collaboration network, visualizing relationships among institutions that have published at least two papers in the field. This network consists of 79 institutions grouped into 25 clusters based on their level of collaboration. Table 2 summarizes the top 10 institutions ranked by publication output. Among them, the Korea Institute of Oriental Medicine and the University of Duisburg-Essen led the field with 13 publications each. China’s contribution was highlighted by the Beijing University of Chinese Medicine, which published 10 papers with an ACPP of 24. Other contributors include the University of Tromsø in Norway, producing 8 publications with an ACPP of 36.25, and the Ministry of Health in Saudi Arabia, which published 6 papers with an ACPP of 42. The institutional collaboration network revealed dense connections between institutions from South Korea. The Korea Institute of Oriental Medicine formed the central hub of collaboration, linking strongly with other South Korean institutions such as the Pusan National University and Kyung Hee University. The University of Duisburg-Essen also demonstrated significant connections with international partners, emphasizing its role in fostering global research networks.

**TABLE 2:** Top 10 institutions ranked by number of publications.

Rank	Institutions	Publications	TC	ACPP	TLS	Location
1	Korea Institute of Oriental Medicine	13	169	13	29	South Korea
2	University of Duisburg-Essen	13	571	43.92	12	Germany
3	Pusan National University	12	130	10.83	28	South Korea
4	Kyung Hee University	11	117	10.64	27	South Korea
5	Beijing University of Chinese Medicine	10	240	24	6	China
6	University of Tromsø	8	290	36.25	10	Norway
7	Wonkwang University	6	17	2.83	15	South Korea
8	Gachon University	6	37	5.67	14	South Korea
9	Ministry of Health—Saudi Arabia	6	252	42	3	Saudi Arabia
10	Guangzhou University of Chinese Medicine	6	69	11.5	1	China

TC, total citations; ACPP, average number of citations per publication; TLS, total link strength.

### 3.3 Analysis of authors

**FIGURE 4:**
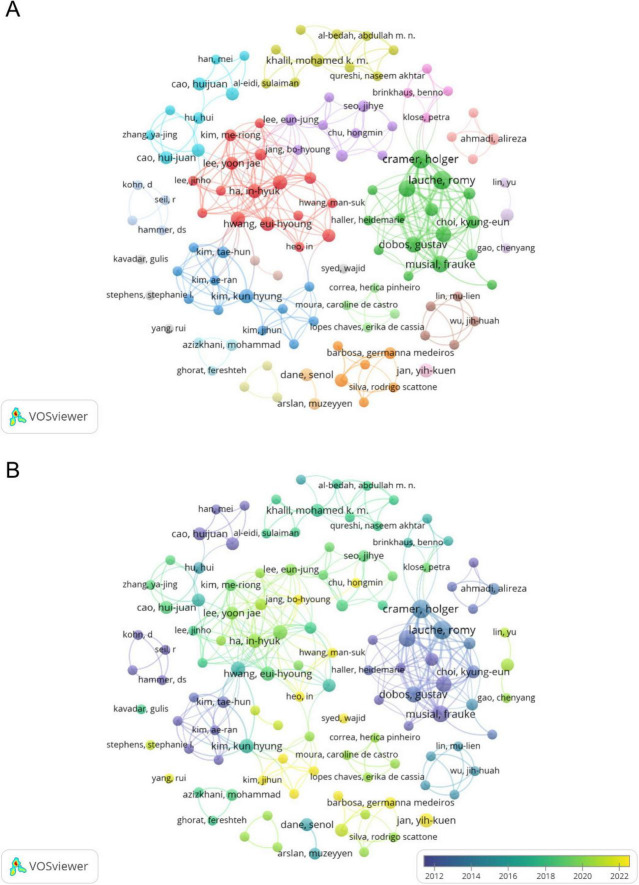
**(A)** Collaborative network knowledge map of author. **(B)** Temporal map of author’s collaborative network.

A total of 1,172 authors have contributed to this research field. Table 3 presents the top 10 authors ranked by publication output. This study analyzed contributions from multiple authors in the field of CT research for pain. Among them, Dobos G leads with 11 publications, 559 TC, 54 TLS, and 50 H-index. He is followed by Cramer H, who has 10 publications, 447 TC, 51 TLS, and 52 H-index. Lauche R ranks third with 10 publications, 455 TC. Other prominent contributors include Langhorst J, Musial F, and Liu JP, all of whom have achieved high citations and high H-index values (61). The author collaboration network is shown in Figure 4A, where nodes represent individual authors, and the size of each node correlates with their number of publications. The connections between nodes signify collaboration strength, with thicker lines indicating stronger partnerships. The network reveals a dense cluster of authors from Germany, including Dobos G, Cramer H, Lauche R, and Musial F, reflecting a strong collaborative environment among German researchers. Similarly, contributors from South Korea, such as Ha IH and Lee MS, form another active cluster. Chinese researchers, including Liu JP and Cao HJ, are also embedded in collaborative networks, demonstrating their integration into global research efforts. Figure 4B maps the temporal evolution of collaborations, represented by color gradients. The darker colors correspond to earlier contributions, while the lighter colors indicate recent activity. German authors have consistently contributed to CT research for pain over the years, demonstrating sustained leadership. In contrast, South Korean and Chinese authors show increased engagement in recent years.

**TABLE 3:** Top 10 authors ranked by number of publications.

Rank	Authors	Publications	TC	ACPP	TLS	Location	H-index
1	Dobos G	11(4.70%)	559	50.82	54	Germany	50
2	Cramer H	10(4.26%)	447	44.7	51	Germany	52
3	Lauche R	10(4.26%)	455	45.5	48	Germany	47
4	Langhorst J	8(3.40%)	403	50.38	41	Germany	56
5	Musial F	8(3.40%)	351	43.88	37	Germany	26
6	Liu JP	8(3.40%)	227	28.38	21	China	61
7	Cao HJ	8(3.40%)	215	26.88	18	China	30
8	Lee MS	6(2.55%)	109	18.17	23	South Korea	57
9	Rampp T	6(2.98%)	346	57.67	38	Germany	15
10	Ha IH	5(2.13%)	37	7.4	30	South Korea	24

TC, total citations; ACPP, average number of citations per publication; TLS, total link strength.

### 3.4 Analysis of journals

**FIGURE 5:**
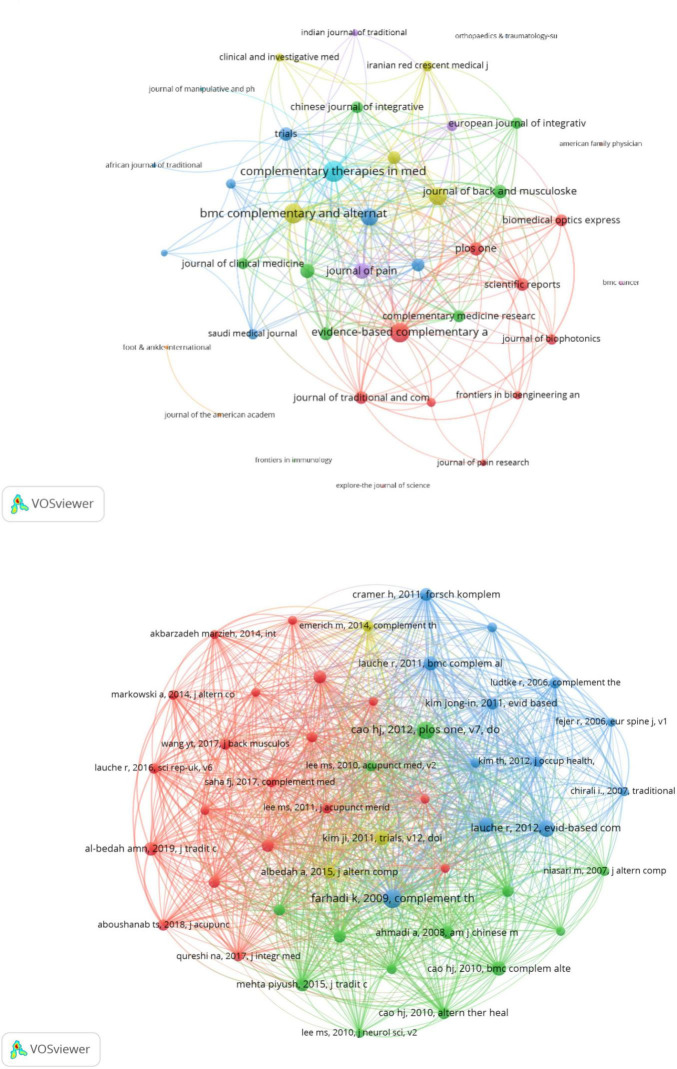
**(A)** Network analysis of collaboration between contributing journals. **(B)** References co-cited network knowledge map.

The analysis of journals revealed that the research landscape of CT for pain has been influenced by the findings presented in these publications. Table 4 show the top 10 journals in this field in terms of total number of publications and TC, respectively. *Evidence-Based Complementary and Alternative Medicine* has published 14 publications with 276 TC. *Complementary Therapies in Medicine* was ranked second, with 10 articles and the highest TC (356). This journal also demonstrated a significant impact factor (IF 3.3), ranking in Q1 (JCR). Figure 5A shows dense connections in the journal collaboration network, with *Complementary Therapies in Medicine*, *BMC Complementary and Alternative Medicine*, and *Evidence-Based Complementary and Alternative Medicine* serving as central nodes. These journals represent pivotal platforms for disseminating studies on CT for pain.

**TABLE 4:** Top 10 journals ranked by number of publications.

Rank	Journals	Publications	TC	TLS	JCR	IF(2024)
1	Evidence-Based Complementary and Alternative Medicine	14	276	86	Q3	2.6
2	Complementary Therapies in Medicine	10	356	109	Q1	3.3
3	European Journal of Integrative Medicine	10	43	19	Q3	1.9
4	Medicine	9	27	27	Q2	1.3
5	Forschende Komplementärmedizin	8	178	69	Q4	1.3
6	Journal of Alternative and Complementary Medicine	6	183	62	Q2	2.3
7	PLoS ONE	6	339	34	Q1	2.9
8	BMC Complementary and Alternative Medicine	5	219	88	Q1	3.3
9	Complementary Therapies in Clinical Practice	5	63	29	Q2	2.2
10	Journal of Traditional Chinese Medicine	5	42	14	Q2	2

TC, total citations; ACPP, average number of citations per publication; TLS, total link strength; IF, impact factor.

### 3.5 Analysis of co-citation references

Figure 5B presents the co-citation networks of publications that have been cited at least 10 times, 47 publications, offering a clear representation of the co-citation relationships among these articles. Table 5 provides an overview of the top 10 most frequently cited references, showcasing the foundational and contemporary trends in this field. These references highlight the evolving understanding of the mechanisms, efficacy, and applications of CT for pain. The 10 most co-cited references include a mix of original research articles (6) and review articles (4). The references span a timeframe from 2008 to 2018. The content of these references can be classified into three primary categories: clinical efficacy trials, exploration of mechanisms, and systematic reviews and comprehensive evaluations.

**TABLE 5:** Top 10 co-cited references ranked by total citations.

Rank	Title	First author	Year	Type	TC	TLS
1	The effectiveness of wet-cupping for non-specific low back pain in Iran: A randomized controlled trial	Khosro Farhadi	2009	Article	46	361
2	An updated review of the efficacy of cupping therapy	Huijuan Cao	2012	Review	40	291
3	The effect of traditional cupping on pain and mechanical thresholds in patients with chronic non-specific neck pain: a randomized controlled pilot study	Romy Lauche	2012	Article	35	309
4	Effects of traditional cupping therapy in patients with carpal tunnel syndrome: a randomized controlled trial	Andreas Michalsen	2009	Article	28	241
5	Evaluation of wet-cupping therapy for persistent non-specific low back pain: a randomized, waiting-list controlled, open-label, parallel-group pilot trial	Jong-In Kim	2011	Article	27	245
6	Clinical research evidence of cupping therapy in China: a systematic literature review	Huijuan Cao	2010	Review	27	189
7	The influence of a series of five dry cupping treatments on pain and mechanical thresholds in patients with chronic non-specific neck pain—a randomized controlled pilot study	Romy Lauche	2011	Article	25	238
8	Cupping therapy: A prudent remedy for a plethora of medical ailments	Piyush Mehta	2015	Review	24	207
9	The medical perspective of cupping therapy: Effects and mechanisms of action	Al-Bedah AMN	2018	review	23	172
10	The efficacy of wet-cupping in the treatment of tension and migraine headache	Alireza Ahmadi	2008	article	22	173

TC, total citations; TLS, total link strength.

### 3.6 Analysis of keywords

**FIGURE 6:**
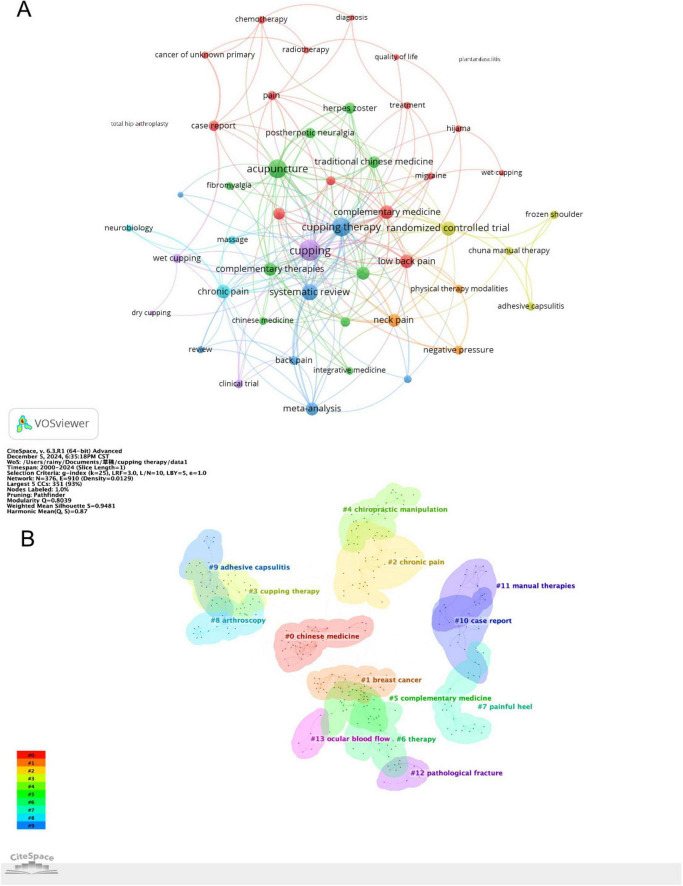
**(A)** Keywords co-occurrence network knowledge map. **(B)** Cluster map of keywords.

**FIGURE 7:**
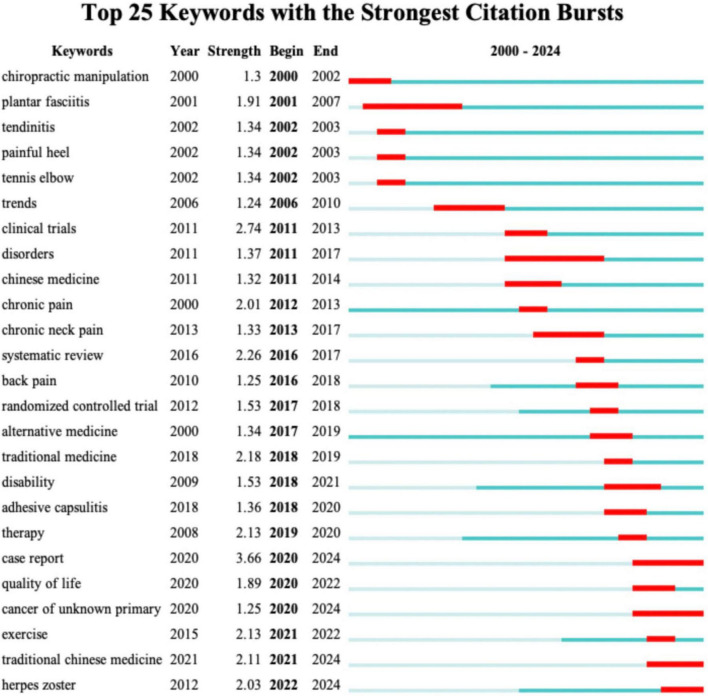
The top 25 keywords with the highest burst strength.

The analysis of keywords provides critical insights into the research focus and emerging trends within the field of CT for pain. Figure 6A presents the co-occurrence networks map of keyword with at least 3 occurrences. As shown in Table 6, the top 40 high frequency keywords highlight the research focus within the field of CT for pain. “Cupping therapy,” “acupuncture,” “chuna manual therapy,” “wet cupping” and “acupuncture” underscore the central role of traditional therapeutic practices in this field. Pain-related keywords, including “chronic pain,” “low back pain,” “adhesive capsulitis,” “knee osteoarthritis,” “herpes zoster,” “migraine” and “neck pain,” “fibromyalgia” indicate the primary conditions targeted by CT, reflecting its application in musculoskeletal and chronic pain relief. These keywords, “Korean medicine,” “traditional medicine,” “traditional Chinese medicine,” “integrative medicine,” “complementary medicine,” “alternative medicine,” and “integrative medicine,” emphasize the globalization and cross-cultural acceptance of traditional therapies. They illustrate how practices originating in specific cultural contexts are being studied, adapted, and integrated worldwide. As demonstrated in Figure 6B, a cluster map of co-cited references has been generated using CiteSpace. The modularity Q value of 0.8039 and the mean silhouette value of 0.9481 confirm the robustness of the clustering, as both metrics surpass the standard threshold of 0.5 (16). Each circle signifies a keyword, with circles of the same color forming cohesive groups around shared themes, identifying 14 distinct clusters. By segmenting the timeline into 1-year intervals, the top 25 keywords with the highest burst intensities were identified, as shown in Figure 7. The keyword “chiropractic manipulation” emerged first, while “plantar fasciitis” exhibited the longest burst. Notably, terms like “traditional Chinese medicine” and “herpes zoster” are still experiencing active bursts. Analyzing these bursts provides a clearer understanding of research hotspots and emerging trends, offering direction for future investigations.

**TABLE 6:** Top 40 keywords ranked by occurrence frequencies.

Rank	Keywords	Occurrence	TLS	Rank	Keywords	Occurrence	TLS
1	Cupping	38	60	21	Herbal medicine	4	10
2	Cupping therapy	29	43	22	Clinical trial	4	7
3	Acupuncture	24	45	23	Back pain	3	9
4	Systematic review	14	30	24	Negative pressure	3	8
5	Chronic pain	12	22	25	Adhesive capsulitis	3	7
6	Complementary medicine	12	22	26	Chuna manual therapy	3	7
7	Low back pain	12	20	27	Frozen shoulder	3	7
8	Randomized controlled trial	11	24	28	Massage	3	7
9	Case report	11	11	29	Chemotherapy	3	6
10	Neck pain	10	19	30	Chinese medicine	3	6
11	Traditional Chinese medicine	8	15	31	Fibromyalgia	3	6
12	Meta-analysis	7	18	32	Integrative medicine	3	6
13	Pain	7	9	33	Knee osteoarthritis	3	6
14	Alternative medicine	6	19	34	Cancer of unknown primary	3	5
15	Traditional medicine	6	14	35	Hijama	3	5
16	Complementary therapies	5	18	36	Neurobiology	3	5
17	Herpes zoster	5	13	37	Review	3	5
18	Korean medicine	5	8	38	Treatment	3	5
19	Wet cupping	5	8	39	Radiotherapy	3	4
20	Migraine	5	5	40	Diagnosis	3	3

TLS, total link strength.

## 4 Discussion

### 4.1 Analysis of current research status

The bibliometric analysis of CT research for pain reveals an evolving field, marked by increasing scholarly engagement and global collaboration. Over the past decades, the steady upward trajectory of research output highlights an intensified focus on non-pharmacological approaches to pain management. This growth aligns with the global emphasis on complementary and integrative medicine, as healthcare systems worldwide strive to address the limitations of conventional pain treatments, including opioid dependency and adverse side effects (17, 18). The observed increase in citations can be attributed to the growing influence and quality of research in this field. A steady growth in annual TC, particularly in recent years, indicates that CT has attracted the attention of researchers from various disciplines, including clinical medicine, physiology, and alternative medicine. This trend suggests that CT has moved beyond anecdotal or tradition-based practices and entered the realm of rigorous scientific validation.

The distribution of publications across countries and institutions provides valuable insights into the global dynamics in CT research for pain. Notably, China, the United States, South Korea, and Germany have emerged as pivotal contributors, reflecting a diverse blend of cultural heritage, scientific curiosity, and modern healthcare imperatives. China has the highest publication volume, which highlights its extensive history in Traditional Chinese Medicine and its substantial governmental support for research into indigenous practices. Nevertheless, the comparatively lower ACPP in China suggests potential obstacles to achieving broader global resonance and scientific impact. These limitations may stem from methodological constraints or an emphasis on local clinical relevance over global applicability. The United States and Germany maintain a smaller but high-impact research output. This reflects their emphasis on rigorous methodologies, robust peer review standards, and evidence-based frameworks, which are critical for the global acceptance and integration of CT into mainstream medicine. The higher ACPP in these countries may also be attributed to their multidisciplinary approaches, integrating CT research with fields such as neurology, chronic pain management, and rehabilitation science. This integration of diverse fields is likely to enhance the credibility and utility of their research in a variety of clinical contexts. The dominance of academic institutions in driving research underscores the importance of higher education and research infrastructure in shaping the field. Leading institutions, such as the University of Duisburg-Essen in Germany and Beijing University of Chinese Medicine in China, serve as hubs of innovation and collaboration, linking academic inquiry with clinical applications. Institutions such as the Korea Institute of Oriental Medicine play a central role in fostering both academic collaborations and clinical applications, further establishing South Korea as a leader in evidence-based traditional medicine. The dense institutional networks within South Korea reflect a coordinated approach to research, which can serve as a model for other countries aiming to scale their scientific efforts in complementary and alternative medicine. Moreover, the integration of local cultural practices with global scientific standards can ensure that research outcomes are both culturally sensitive and scientifically robust.

A prominent feature of CT research for pain is the significant contribution of collaborative author teams, which have emerged as central to the field’s development. Scholars such as Dobos G, Cramer H, Lauche R, Langhorst J, and Musial F, hailing from the University of Duisburg-Essen in Germany, have devoted their research to examining the clinical and mechanistic effects of CT on chronic non-specific neck pain management. For instance, their seminal study explored the efficacy of traditional wet CT for chronic non-specific neck pain and its effects on mechanical sensory thresholds. This randomized controlled trial (19) demonstrated that wet CT led to significant pain reduction, improved functional outcomes, and altered mechanical detection, vibration detection and pressure pain thresholds, suggesting both central and peripheral mechanisms of action. In some other similar studies, they investigated the effects of CT on chronic non-specific neck pain. Their findings (20, 21) revealed significant improvements in pain intensity, pressure pain sensitivity, functional disability, mental health, the mental component summary, and the quality-of-life subscales pain compared to control groups. Additionally, the team (22) explored dry CT’s impact on fibromyalgia syndrome, finding that while dry CT was more effective than empty control group for reducing pain and enhancing quality of life, its effects were small and similar to sham treatment. These works collectively highlight the diverse applications and outcomes associated with CT in pain conditions. Similarly, the team led by Liu JP and Cao HJ, primarily based at Beijing University of Chinese Medicine, has significantly contributed to CT research for pain. Their studies have provided a comprehensive overview of CT’s clinical effectiveness. For instance, their study on CT has demonstrated its potential to reduce pain and tender points in fibromyalgia patients, with effects sustained beyond the treatment period. Additionally, a systematic review (23) of traditional Chinese medicine therapies, including acupuncture, cupping, and herbal medicine, demonstrated effects in reducing pain and improving symptoms in fibromyalgia, but highlighted methodological limitations. Similarly, a comparative review (23) of CT and acupuncture for pain-related conditions showed that both therapies are potentially safe and have similar effectiveness in relieving pain across conditions like cervical spondylosis, lateral femoral cutaneous neuritis, and scapulohumeral periarthritis. In South Korea, Lee MS and Ha IH, both from the Korea Institute of Oriental Medicine, have become prominent figures in integrating CT into modern healthcare systems. Their research has made important contributions to understanding the effectiveness and safety of CT. For example, Lee MS conducted a pilot study (24) on wet CT for persistent non-specific low back pain, showing its potential to lower pain intensity and reduce the use of acetaminophen. Ha IH led a study (25) on the cost-effectiveness of integrated therapies, including CT, for treating neck pain caused by traffic accidents. This study demonstrated enhancements in health-related quality of life. A systematic review (26) of adverse events related to CT was also conducted. These efforts underscore the importance of evidence-based approaches in safely and effectively incorporating CT into clinical practice. This work highlighted the rarity of such events and emphasized the importance of qualified practitioners and adherence to safety guidelines to minimize risks. These research teams exemplify the importance of interdisciplinary collaboration and international partnerships in advancing CT research for pain.

The analysis of journals publishing research on CT for pain reveals key insights into the dissemination and focus of scientific work in this field. High-impact journals such as *Journal of Alternative and Complementary Medicine*, *Complementary Therapies in Medicine*, and *BMC Complementary Medicine and Therapies* emerge as leading platforms for publishing evidence-based research on complementary and integrative medicine. These journals, renowned for their rigorous peer-review processes, highlight the growing scientific interest in validating traditional practices like CT. Among these, *Complementary Therapies in Medicine* has played a pivotal role, publications that explore the broader implications of CT within integrative medicine. Its focus on both clinical and mechanistic studies reflects a balanced approach to understanding the utility and safety of CT.

### 4.2 Analysis of research hotspots and trends

Building on the insights gained from co-citation reference analysis and keyword mapping, this section delves into the pivotal research areas and emerging trends in CT for pain. By synthesizing the patterns and themes identified, we explore 4 key dimensions: mechanisms of CT for pain, therapeutic efficacy of CT for pain, combined applications of CT for Pain, and future trends in CT research. This structured approach aims to provide a comprehensive understanding of the current landscape while highlighting areas ripe for further exploration.

Mechanisms of CT for pain: CT provides a multifaceted approach to relieving pain by targeting biomechanical, neurological, and immunological processes (27). At its core, CT improves microcirculation by creating negative pressure on the skin, which draws blood to the treated area. This increases capillary expansion and accelerates the removal of metabolic waste and inflammatory mediators. The enhanced blood flow delivers oxygen and nutrients to damaged tissues while also helping to clear harmful substances that contribute to inflammation and pain (1, 28–30). CT may be able to stimulate sensory afferent nerves, activating the body’s natural pain modulation pathways. This mechanical stimulus aligns with the gate control theory of pain, where non-painful stimuli reduce the transmission of painful signals, thereby lowering the perception of pain (31). Additionally, CT has been found to promote the release of endogenous opioids, serotonin, and other neuromodulators, which enhance its systemic analgesic effects (32, 33). Another role of CT is to treat myofascial pain syndrome, a common cause of chronic pain, by releasing fascial adhesions. By stretching the fascia and relieving tension, CT restores normal mobility to the tissues and reduces localized discomfort (34). From an immunological perspective, CT modulates the inflammatory response by reducing pro-inflammatory cytokines such as IL-6 and TNF-α, while simultaneously increasing anti-inflammatory mediators (9, 28, 35). This dual action helps to reduce local and systemic inflammation, addressing a key factor in chronic pain. In summary, these mechanisms not only alleviate pain but also promote long-term functional recovery, providing an evidence-based, integrated approach to pain management.

Therapeutic efficacy of CT for pain: The therapeutic efficacy of CT in managing various pain conditions has been consistently validated through several clinical trials. One of the most notable areas is its application in chronic non-specific pain conditions, such as low back pain and neck pain, which significantly impact quality of life. In these conditions, CT has demonstrated measurable improvements in pain intensity, functional mobility, and patient-reported outcomes. For instance, several studies (4, 36–38), highlighted the effectiveness of CT in reducing chronic non-specific pain with patients reporting significant reductions in pain severity and enhanced sensory thresholds. These findings underscore CT’s capacity to address the multifactorial nature of chronic pain by targeting both the mechanical and neurological contributors. Beyond musculoskeletal disorders, CT has been increasingly employed to alleviate headaches and migraines, conditions often associated with vascular and muscular dysfunction. By improving cervical mobility and reducing tension in cranial and upper cervical muscles, CT provides relief from tension-type headaches and migraines (8, 39, 40). Furthermore, CT serves as an adjunct to conventional pain management protocols, demonstrating its potential to reduce reliance on opioid analgesics (6, 32). CT has also been shown to be a modality for the management of inflammatory conditions, including osteoarthritis and rheumatoid arthritis (7, 35, 41). For scoliosis, CT has been investigated as a complementary approach to reduce muscle stiffness and improve spinal mobility, thereby alleviating discomfort associated with the condition (42). For ankylosing spondylitis, CT has demonstrated promise in reducing pain and inflammatory markers, offering symptomatic relief and enhancing the overall quality of life for patients with this debilitating autoimmune disease (43). Similarly, for carpal tunnel syndrome, CT has been employed to improve nerve conduction, reduce localized inflammation, and alleviate pain and numbness in affected hands, offering an alternative to invasive surgical procedures (44–47).

Combined applications of CT for pain: The potential for combining CT with other therapeutic modalities represents a promising frontier in the field of integrative pain management. One of the most studied combinations is CT with acupuncture, a pairing that enhances the analgesic effects of both interventions. The combination of CT and acupuncture has been shown to produce favorable outcomes in the treatment of conditions such as acute herpes zoster (48, 49), postherpetic neuralgia (50, 51) and chronic low back pain (31, 52). This is attributed to the enhanced microcirculatory improvement and neuromodulatory effects of the combined approach. Patients who receive both therapies may experience faster and longer-lasting pain relief than those who receive only one of the therapies (53). Similarly, the incorporation of CT into physical therapy protocols has yielded favorable outcomes. When combined with stretching and strengthening exercises, CT addresses both the structural and functional components of pain, thereby facilitating comprehensive recovery (54, 55). Another application is the combination of CT with herbal medicine and acupuncture, which is a common practice in traditional medicine systems. The combination of these three therapies enhances the anti-inflammatory effects of CT, as the herbal components complement CT’s ability to reduce localized inflammation and improve circulation (56–59). The recent integration of CT with dry needling techniques provides further evidence of its versatility. This approach has demonstrated efficacy in the treatment of myofascial pain syndromes, with CT facilitating improved blood flow and dry needling targeting trigger points (60–62). Such combinations not only maximize therapeutic outcomes but also reflect a growing trend toward personalized medicine. By adapting treatments to the specific needs of each patient, healthcare professionals can enhance the efficacy of CT and address the complex nature of chronic pain.

Future trends in CT research for pain: As the field of CT continues to develop, several emerging trends and challenges underscore the need for continued research and innovation. One key area for future research is the standardization of CT protocols. The standardization of CT trials can be significantly improved by adhering to the Standards for Reporting Interventions in Clinical Trials of Cupping (STRICTOC), an extension of the CONSORT guidelines. The STRICTOC checklist provides comprehensive guidance across 6 domains, encompassing 16 sub-items that detail crucial aspects of cupping intervention. These include clearly articulating the rationale for CT, providing explicit details of the cupping devices used (e.g., cup materials like glass or plastic, device size typically ranging from 2.5 to 6 cm in diameter, manufacturers), and specifying the method for generating negative pressure. Manual suction, fire cupping, or mechanical pumps are most often applied at around –70 mbar for mild intensity and –150 to –400 mbar for stronger applications, although values exceeding –500 mbar have been reported for certain anatomical sites (63). Furthermore, it emphasizes clear reporting of anatomical locations (specific acupuncture points or anatomical landmarks), duration of each cupping session (generally between 5 and 15 min per site), frequency (1–3 sessions weekly), and total number of sessions (commonly between 3 and 12 over several weeks). STRICTOC also addresses important safety measures, treatment provider qualifications and experience, and precise details of any control or comparator interventions employed (64). Achieving standardization in these parameters will be essential to enhance CT’s credibility and ensure its consistent and effective application in clinical settings. The integration of advanced technologies into CT represents another promising avenue for future research. For example, a study (30) using embedded near-infrared spectroscopy monitors has demonstrated the impact of CT on microcirculation and tissue perfusion by revealing changes in hemodynamic parameters during treatment. Similarly, optical imaging modalities such as photoacoustic imaging have been instrumental in quantifying microenvironmental changes during CT sessions. These technologies have been used to evaluate CT efficacy under varying pressures, showing improvements in tissue oxygenation and vascular dynamics (1, 65). Additionally, ultrasound imaging has offered new insights into CT’s therapeutic effects, with texture analysis revealing enhancements in muscle quality, such as in the triceps, after CT treatments. Research on muscle stiffness using ultrasound imaging has also highlighted the role of treatment duration and pressure in reducing stiffness and restoring normal muscle function (66, 67). Emerging quantitative tools such as shear-wave elastography (SWE) and multichannel surface electromyography (sEMG) are now being adopted to characterize muscular responses to cupping therapy. For example, a RCT is using SWE to track changes in lumbar-paraspinal stiffness after 2 weeks of moving cupping in 68 participants with CLBP (68). Likewise, a recent RCT combined acupuncture with a single flash-cupping session and demonstrated improved horizontal synchronization of paraspinal muscle activity on sEMG topographic mapping, despite no immediate difference in pain scores (69). These modalities provide objective endpoints that complement subjective pain scales. By incorporating these technologies, researchers can deepen their understanding of CT’s underlying mechanisms toward standardizing treatment protocols based on measurable outcomes. Furthermore, global collaboration will be essential to validate the efficacy of CT across diverse populations and healthcare systems. Expanding research efforts beyond traditional strongholds such as China and South Korea will ensure that findings are generalizable and culturally inclusive. By addressing these areas, CT research can continue to bridge the gap between traditional practices and modern medicine, solidifying its role in the future of integrative pain management.

### 4.3 Limitations

This study has certain limitations that need to be acknowledged. First, the bibliometric analysis was confined to publications indexed in the WoSCC database. Consequently, Chinese-language trials in CNKI or Wanfang, Korean and Persian cupping therapy studies indexed only in regional databases, and gray literature such as dissertations or conference papers were not captured, so some clinically relevant evidence may have been missed. Second, the analysis was restricted to English-language publications, thereby excluding important contributions published in other languages, particularly those from countries where CT is deeply rooted in traditional medicine. Non-English case series and small randomized trials from Middle-Eastern journals that focus on hijama, for example, were likely overlooked, which could limit the cultural breadth of our conclusions. Third, the bibliometric tools employed, such as VOSviewer and CiteSpace, utilize predefined algorithms and parameters, which introduce potential biases in network clustering, co-citation mapping, and keyword analyses. Finally, while this study identifies research hotspots and trends, it does not thoroughly assess the quality or clinical impact of individual studies. Future analyses should aim to incorporate a more diverse dataset, include quality assessments of studies, and explore broader cultural and linguistic contexts to provide a more holistic view of the field.

## 5 Conclusion

This bibliometric analysis provides a comprehensive examination of the research landscape in CT research for pain. Over the past decades, the publications and citations in this field has shown steady growth. The analysis underscores key contributors, including leading countries such as China, the United States, South Korea, and Germany, have established themselves as research center in advancing the understanding and application of CT. Institutions like the Korea Institute of Oriental Medicine, Beijing University of Chinese Medicine, and the University of Duisburg-Essen, along with prominent researchers such as Dobos G, Liu JP, and Lee MS, have significantly influenced the field through their high-quality studies and international collaborations. From the analysis of research trends, several key insights emerged. The therapeutic efficacy of CT for various pain conditions, including neck pain and low back pain, has been substantiated by several studies. The growing integration of CT with other therapies, such as acupuncture, physical therapy, and pharmacological interventions, emphasizes trend toward multimodal approaches in pain management. Despite its advancements, the field faces challenges such as the need for standardized protocols, deeper mechanistic understanding, and high-quality clinical trials. In summary, this study identifies CT as a potential tool in pain management, with progress made in understanding its mechanisms, efficacy, and applications. By bridging traditional practices with modern scientific methodologies, CT research is poised to make contributions to integrative medicine, offering new avenues for effective, evidence-based pain management strategies.

## Data Availability

The datasets presented in this study can be found in online repositories. The names of the repository/repositories and accession number(s) can be found in the article/supplementary material.
